# Ginger in gastrointestinal disorders: A systematic review of clinical trials

**DOI:** 10.1002/fsn3.807

**Published:** 2018-11-05

**Authors:** Mehrnaz Nikkhah Bodagh, Iradj Maleki, Azita Hekmatdoost

**Affiliations:** ^1^ Student Research Committee Faculty of Nutrition and Food Technology Shahid Beheshti University of Medical Sciences Tehran Iran; ^2^ Gut and Liver Research Center Mazandaran University of Medical Sciences Sari Iran; ^3^ Department of Clinical Nutrition and Dietetics Faculty of Nutrition Sciences and Food Technology National Nutrition and Food Technology Research Institute Shahid Beheshti University of Medical Sciences Tehran Iran

**Keywords:** dyspepsia, fatty liver, gastrointestinal cancer, gastrointestinal mucosa, gastroprotective, ginger, gingerol, irritable bowel syndrome, nausea, shogaol, swallowing, vomiting gastric emptying, Zerumbone, Zingiber

## Abstract

Ginger, the rhizome of Zingiber officinale, which is used as a spice globally has a long history of medicinal use that stimulates investigators to assess its potential roles as an adjuvant therapy or alternative medicine in a range of diseases. Anti‐inflammatory, antioxidant, antitumor, and antiulcer effects of ginger have been proven in many scientific studies, and some of the ancient applications of ginger as a home remedy has been confirmed in human. In this review, we summarized the current evidence on the effects of ginger consumption on gastrointestinal disorders based on clinical trials. Our data indicate that divided lower daily dosage of 1500 mg ginger is beneficial for nausea relief. Because of limited number of studies on some other gastrointestinal disorders, the results may not be as much powered as to find significant results. Therefore, more extensive and well‐controlled human studies of ginger or its standard extracts are required to demonstrate its efficacy as a gastroprotective agent. Dose‐finding studies should be undertaken to accurately determine the effective dose and preparation of ginger in further clinical trials protocol.

## 1 INTRODUCTION

Ginger, the rhizome of Zingiber officinale, is a member of the Zingiberaceae family that has been used as a spice globally. This spice contains a wide variety of volatile and nonvolatile compounds with various concentrations depending on different condition of cultivation, harvesting, and processing (Haniadka et al., 2013). Chemical analysis of ginger shows that it contains over 400 different compounds. The major constituents in ginger rhizomes are carbohydrates (50–70%), lipids (3–8%), terpenes, and phenolic compounds (Grzanna, Lindmark, & Frondoza, 2005). Terpene components of ginger include zingiberene, β‐bisabolene, α‐farnesene, β‐sesquiphellandrene, and α‐curcumene, while phenolic compounds include gingerol, paradols, and shogaol. The specific odor of ginger is related to zingiberene and bisabolene, while the pungent flavor is due to volatile oils of gingerols (23–25%) and shogaols (18–25%). Besides these components, amino acids, raw fiber, ash, protein, phytosterols, vitamins (e.g., nicotinic acid and vitamin A), and minerals are also present in ginger. Other gingerol‐ or shogaol‐related compounds (1–10%), which have been reported in ginger rhizome, include 6‐paradol, 1‐dehydrogingerdione, 6‐ gingerdione and 10‐gingerdione, 4‐ gingerdiol, 6‐gingerdiol, 8‐ gingerdiol, and 10‐gingerdiol, and diarylheptanoids (Ali, Blunden, Tanira, & Nemmar, 2008; McGee, 2007; Prasad & Tyagi, 2015).

Since thousands of years ago, ginger has been used as a food and herbal medicine in Asia and the Far East so that its medical use is well described in Chinese remedies from 400 BC. The rhizomes have been used since antiquity in the various traditional systems of medicine to treat cold, fever, sore throats, infectious diseases, arthritis, rheumatism, sprains, muscular aches, pains, cramps, hypertension, dementia, migraine, nervous diseases, gingivitis, toothache, asthma, stroke, and diabetes and also used as home remedy in treating various gastric ailments like constipation, diarrhea, dyspepsia, belching, bloating, gastritis, epigastric discomfort, gastric ulcerations, indigestion, nausea, and vomiting (Giacosa, Morazzoni, et al., 2015; Haniadka et al., 2013; Lete & Allué, 2016). This long and established history of medicinal use of ginger in humans has stimulated clinical trials to scientifically assess the effectiveness of ginger as an adjuvant therapy or as a complementary and alternative medicine in a number of diseases especially gastrointestinal ailments (Lete & Allué, 2016). Anti‐inflammatory, antioxidant, antitumor, and antiulcer effects of ginger have been proven in some studies; however, some results are controversial, probably due to the chemical instability of gingerols (the ginger most active ingredients), which are readily oxidizable substances (Giacosa, Morazzoni, et al., 2015; Lete & Allué, 2016). The U.S. Food and Drug Administration classifies ginger as “Generally Recognized as Safe” and the German Commission Monographs reported that ginger has no known side effects and no known drug/herb interactions (Blumenthal, 1998). In this review, we summarize recent studies evaluating the effects of ginger consumption in gastrointestinal disorders.

## 2 GASTRIC EMPTYING AND DYSPEPSIA

Ginger as an important dietary agent which possesses carminative effect, decreases pressure on lower esophageal sphincter, reduces intestinal cramping, and prevents dyspepsia, flatulence, and bloating (Ali et al., 2008; Chrubasik, Pittler, & Roufogalis, 2005; Lohsiriwat, Rukkiat, Chaikomin, & Leelakusolvong, 2010). A clinical trial (Giacosa, Morazzoni, et al., 2015) investigated the effects of ginger extract (100 mg, corresponding to 2 g of rhizome twice a day) on gastrointestinal motility and showed a significant increase in gastrointestinal motility in the intervention group in comparison with the placebo (Micklefield et al., 1999). Wu et al. (2008) showed that ginger accelerates gastric emptying and stimulates antral contractions in healthy individuals; studies on patients with functional dyspepsia have shown the same results with no alterations in the fundus dimension, gastrointestinal symptoms, or serum level of gut peptides such as GLP‐1, motilin, and ghrelin (Hu et al., 2011).

Impaired gastric emptying is a well‐recognized contributor to the pathophysiology of gastrointestinal problems such as functional dyspepsia and nausea. Functional dyspepsia is defined as postprandial fullness, early satiety, or epigastric pain/burning or discomfort centered in the upper abdomen in the absence of any known structural cause and without features of irritable bowel syndrome or gastroesophageal reflux; symptoms are frequently correlated to meals and may include abdominal pain, bloating, early satiety, fullness, belching, and nausea (Talley et al., 1999). Prodigest is the standardized combination of ginger extracts and artichoke extract. Artichoke completes the effects of ginger because ginger is active on stomach while artichoke on small bowel. A randomized, 4‐week trial on patients with functional dyspepsia showed that daily consumption of Prodigest before lunch and dinner resulted in a significant amelioration of functional dyspepsia symptoms including nausea, epigastric fullness, epigastric pain, and bloating as compared with placebo (Giacosa, Guido, et al., 2015). A randomized crossover study investigated the effects of Prodigest on gastric emptying in 11 healthy volunteers. In this study, the baseline area of gastric volume was determined by ultrasonography 10 min before the main meal. Then, the subjects were given one Prodigest or placebo capsule and consumed a standardized meal. One hour after the meal, the gastric volume was measured again. The gastric emptying was compared by differences between gastric volumes. Finally, the results indicated that the after‐meal gastric area was significantly smaller, with a −24% difference, following the combination of extracts, as compared with placebo. So, Prodigest significantly promotes gastric emptying in healthy volunteers without being associated with notable adverse effects (Lazzini, Polinelli, Riva, Morazzoni, & Bombardelli, 2016). These effects on gastric emptying are mostly dependent upon the peculiar molecular actions of the ginger extract. Gastric hypomotility involves a temporary dysfunction of the integrated network of cholinergic M3 and serotonergic 5‐ HT3/5‐HT4 receptors. The major chemical constituents of the ginger roots lipophilic extracts such as [6]‐gingerol, [8]‐gingerol, [10]‐gingerol, and [6]‐shogaol do modulate all these receptors (Giacosa, Guido, et al., 2015; Lazzini et al., 2016).

Delayed gastric emptying leads to gastric intolerance to gavages and increases gastric residual volume, vomiting, risk of aspiration, and the length of stay in hospital in 30–51% of the patients (Davies, 2010; Landzinski, Kiser, Fish, Wischmeyer, & MacLaren, 2008). Delayed gastric emptying and gastrointestinal motility disorder are the main gastrointestinal problem in the severely ill patients undergoing mechanical ventilation. A double‐blind randomized controlled clinical trial showed that consumption of 120 mg ginger extract for 4 days significantly lowered mean residual volume of feeding with a standard gavage solution, on the fifth and the sixth days of feeding (Ghochae et al., 2013). Another study also showed that ginger extract increases the amount of tolerated food, and the amount of calorie intake in patients with adults respiratory distress syndrome under mechanical ventilation (Shariatpanahi, Taleban, Mokhtari, & Shahbazi, 2010).

## 3 NAUSEA AND VOMITING

The most common cause of nausea and vomiting is pregnancy. Nausea and vomiting affect up to 80% of women during the first trimester of pregnancy that range from morning sickness to hyperemesis gravidarum (HG) (Lete & Allué, 2016; McCarthy, Lutomski, & Greene, 2014; Palatty, Haniadka, Valder, Arora, & Baliga, 2013). Nonpharmacologic management of nausea in pregnancy includes avoiding nausea‐inducing foods and eating small, frequent meals (Matthews, Dowswell, Haas, & O'Mathúna, 2010). Administration of pyridoxine or doxylamine‐pyridoxine might be beneficial. Medications such as dimenhydrinate, promethazine, ondansetron, or chlorpromazine can be used in severe cases, while 250 mg of ginger taken orally every 6 hr might be added at any time (Arsenault et al., 2002). The impact of ginger consumption as an antiemetic in nausea and vomiting of pregnancy has been extensively investigated in clinical studies for at least 30 years (Giacosa, Morazzoni, et al., 2015). The studies have shown that ginger in dose of 1 g/day is effective in pregnancy nausea and vomiting with no significant side effects (Firouzbakht, Nikpour, Jamali, & Omidvar, 2014; Haji Seid Javadi, Salehi, & Mashrabi, 2013; Saberi, Sadat, Abedzadeh‐Kalahroudi, & Taebi, 2014; Thomson, Corbin, & Leung, 2014; Viljoen, Visser, Koen, & Musekiwa, 2014). American College of Obstetricians and Gynecologists (ACOG), and the National Institute for Health and Clinical Excellence, has accepted ginger as a remedy for nausea and vomiting during early pregnancy (Giacosa, Morazzoni et al., 2015) (Table 1).

**Table 1:** The effects of ginger supplementation on pregnancy‐induced nausea and vomiting

Study ID and date of publication	Type of article	Intervention (ginger dose per day)	Comparator	Duration	Main results
Thomson et al. (2014)	Meta‐analysis (6 studies 1991–2009) (Basirat, Moghadamnia, Kashifard, & Sarifi‐Razavi, 2009; Fischer‐Rasmussen, Kjær, Dahl, & Asping, 1991; Keating & Chez, 2002; Ozgoli, Goli, & Simbar, 2009; Smith, Crowther, Willson, Hotham, & McMillian, 2004; Vutyavanich, Kraisarin, & Ruangsri, 2001)	(<1000 mg daily) 250 mg powder (4×) or 4 times/day (3 studies) 350 mg. 3 times/day 250 mg ginger syr. 4 times/day 500 mg ginger biscuit, five times/day (~2500 mg/day)	Placebo	(4 days–3 weeks)	Ginger was better than placebo in improving nausea when given at a dose of <1000 mg/day for at least 4 days
Viljoen et al. (2014)	Meta‐analysis of 12 studies (1991–2011) 6 additional studies (Chittumma, Kaewkiattikun, & Wiriyasiriwach, 2007; Dante, Pedrielli, Annessi, & Facchinetti, 2013; Ensiyeh & Sakineh, 2009; Mohammadbeigi, Shahgeibi, Soufizadeh, Rezaiie, & Farhadifar, 2011; Pongrojpaw, Somprasit, & Chanthasenanont, 2007; Sripramote & Lekhyananda, 2003) to Thomson et al. (Thomson et al., 2014). Contains reviews by Dante et al. (2013), Ding, Leach, & Bradley (2013)	125 mg extract (4×) /day~1000 mg/day	Placebo	4 days	Ginger was more effective than placebo for reducing nausea and retching. No differences in vomiting
		200 mg essence (3 × )/day ~600 mg/d	Metoclopramide and placebo	5 days	The effects of ginger were not significantly different from metoclopramide
		500 mg powder (2 × )/day~1000 mg/day	Dimenhydrinate	7 days	Ginger to be just as effective as dimenhydrinate, with fewer side effects
		325 mg powder (2×) 3 times/day ~1950 mg/day]	Vitamin B6	4 days	Ginger improved nausea and vomiting symptoms significantly more than B6
		500 mg powder (2×) /day~1000 mg/day		4 days	More effective than B6 for relieving of nausea, equally effective for reduction of vomiting
		500 mg powder (3×) /day~1500 mg/day		3 days	No difference between the two groups
Saberi et al. (2014)	RCT	250 mg ginger capsules three capsules per day	Placebo and control group	7 days (4 days intervention)	Ginger was effective for the relief of mild to moderate nausea and vomiting
Firouzbakht et al. (2014)	RCT	Ginger capsule (Zintoma, 250 mg) one capsule each 6 h~1 g ginger/day	Vitamin B6 and placebo	Treatment for 4 days followed 1 week	Ginger is as effective as B6 in reducing gestational nausea and vomiting
Haji Seid Javadi et al. (2013)	RCT	250 mg ginger capsules (4× daily)	40 mg vitamin B6 capsules (2× daily)	4 days	Ginger was equivalent to vitamin B6 for nausea reduction

Another disturbing cause of nausea and vomiting is the side effects of chemotherapy (Ryan, 2010). Some chemotherapeutic agents, including cyclophosphamide and cisplatin, can lead to a high incidence of nausea and vomiting (Herrstedt & Dombernowsky, 2007). The standard of care for chemotherapy‐induced vomiting is antiemetics, most notably serotonin (5‐HT3) receptor antagonists and glucocorticoids, such as dexamethasone; however, efforts to control nausea have not been successful (Bloechl‐Daum, Deuson, Mavros, Hansen, & Herrstedt, 2006; Herrstedt & Roila, 2008). The summary of studies evaluating the effects of ginger consumption on chemotherapy‐related nausea and vomiting is shown in Table 2 (Ansari et al., 2016; Arslan & Ozdemir, 2015; Fahimi et al., 2011; Manusirivithaya et al., 2004; Marx et al., 2013; Montazeri, Raei, et al., 2013; Panahi et al., 2012; Pillai, Sharma, Gupta, & Bakhshi, 2011; Ryan et al., 2012; Sanaati, Najafi, Kashaninia, & Sadeghi, 2016; Sontakke, Thawani, & Naik, 2003; Thamlikitkul et al., 2017; Zick et al., 2009).

**Table 2:** The effects of ginger supplementation on chemotherapy‐induced nausea and vomiting

Study ID and date of publication	Type of article	Intervention (ginger dose per day)	Comparator	Duration	Main results
Marx et al. (2013)	Systematic review (7 studies 2003–2012) (Fahimi et al., 2011; Manusirivithaya et al., 2004; Panahi et al., 2012; Pillai et al., 2011; Ryan et al., 2012; Sontakke et al., 2003; Zick et al., 2009)	(0.5, 1 or 1.5) g ginger extract/day + antiemetic drug	Placebo + antiemetic drug	Consumption: 2 × 6 day Evaluation: for 3 × 4‐day	All concentrations of ginger significantly reduced the incidence of acute, but not delayed nausea, with 0.5 and 1.0 g being the most effective
		1.5 g (3 × 500 mg)/day + standard antiemetic regimen	Standard antiemetic regimen	Consumption: 4 days from the initiation of chemotherapy evaluation: first 6 hr, between 6 and 24 hr, and days 2, 3, and 4 postchemotherapy	Reduced nausea 6–24 h postchemotherapy, no other significant additional benefit against prevalence or severity of nausea, vomiting, and retching in any of them during the assessed periods.
		1 g ginger (6 × 167 mg) or 2 g (5 × 400 mg) determined by participant's weight	Placebo	Consumption: for 3 days postchemotherapy; evaluation: for 10 days	Reduction in moderate and severe acute and delayed nausea and emesis
		1 g (4 × 250 mg)	Placebo then crossed over	2 × 3‐day (3‐week Washout)	No additional benefit in any measurement of acute or delayed nausea and vomiting to standard control
		1 g (4 × 250 mg or 2 g (8 × 250 mg) per day	Placebo	3 days postchemotherapy	No benefit in any measurement of acute or delayed nausea and vomiting
		1 g ginger (4 × 250 mg)	Placebo crossed over	2 × 5‐day (3–4‐week washout)	No benefit in acute nausea. Reduction in delayed nausea and vomiting equal to standard treatment
		2 g (4 × 500 mg) ginger	2 control groups crossed over	3 × 24‐hr (21 days between Session)	Ginger performed equally as well as metoclopramide in controlling of nausea and vomiting
Arslan & Ozdemir (2015)	Experimental RCT	500 mg powdered ginger, mixed with a spoonful of yogurt (×2)/day + standard antiemetic drugs	Standard antiemetic medicines	30 min before chemotherapy for 3 days followed up for 5 days	Nausea severity and the number of vomiting episodes were significantly lower in the intervention group than control group, the change in the number of retching episodes was not statistically significant
Sanaati et al. (2016)	RCT	500 mg (×2) (~1 g/day) of powdered ginger + routine antiemetic regimen	1‐Matricaria chamomilla extract + routine antiemetic regimen 2‐Control group, routine antiemetic regimen	5 days before and 5 days after chemotherapy	Ginger and chamomile were both significantly effective for reducing the frequency of vomiting; ginger significantly influenced the frequency of nausea
Thamlikitkul et al., (2017)	RCT crossover	500‐mg ginger capsule (×2)/day (~1 g/day) + anti emetics	Placebo+ antiemetics	5 days	There were no significant differences between ginger and placebo in nausea severity, vomiting incidence and severity, rescue medication use, chemotherapy compliance, and adverse events.
Montazeri, Raei, et al. (2013)	RCT crossover	Ginger 1 g (4 × 250 mg)	Placebo	Two chemotherapy cycles	Reduced severity and frequency of nausea and vomiting
Ansari et al. (2016)	RCT	250 mg (×2) ginger powder, (×2)/day (~1 g/day)	Placebo	3 days	There were no significant differences between ginger and placebo in nausea and vomiting except in those patients who received the AC regimen chemotherapy, vomiting was less severe comparing to placebo

Nausea and vomiting can occur after surgery, medications, or even exercise. Despite advances in surgical techniques and introduction of less‐emetogenic anesthetic methods and drugs, postoperative nausea and vomiting, which occurs within 24 hr after surgery, are one of the common and distressing symptoms, following anesthesia and surgery (Palatty et al., 2013). The frequency of upper and lower gastrointestinal disturbance as a consequence of exercise is reported to be between 30 and 70%, with the severity of symptoms ranging from mild stomach discomfort to severe diarrhea (Ball, Ashley, & Stradling, 2015). As it is shown in Table 3, 500 mg ginger per day can reduce postsurgery nausea and vomiting.

**Table 3:** Postoperative or drug‐induced nausea and vomiting

Study ID	Type of article	Cause of nausea and vomiting	Intervention (ginger dose per day)	Comparator	Duration	Main results
Kalava et al. (2013)	RCT	Postoperative and intraoperative nausea and vomiting	Ginger powder 2 g (2 × 1 g) + standard preoperative antiemetic	Placebo standard preoperative antiemetic	Consumption: 1 capsule 30 min before induction of anesthesia and the second 2 hr after surgery evaluation: 0, 2, 2 ½ and 24 hr after surgery	Ginger reduced the number of episodes of intraoperative nausea, but it had no effect on incidence of nausea, vomiting, or pain during and after an elective cesarean section under spinal epidural anesthesia
Mandal et al. (2014)	RCT	Postoperative nausea and vomiting	0.5 g ginger powder (×2)/day ~ 1 g/day + IV Ondansetron (4 mg)	Placebo+ IV Ondansetron (4 mg)	Consumption: 1 hr prior to induction of general anesthesia Evaluation:18 h postoperation	Significantly reduced the incidence of postoperative nausea and vomiting compared to ondansetron alone
Montazeri, Hamidzadeh, et al. (2013)	RCT	Postoperative nausea and vomiting	250 mg ginger powder (×4)	Placebo	Consumption:1 hr before surgery, evaluation: 2, 4, 6 hr postoperation	The frequencies of nausea in the experimental group at 2 hr postoperation were borderline significant but no significant differences between two groups in the intensity of vomiting
Hosseini & Adib‐Hajbaghery (2015)	RCT	After open and laparoscopic nephrectomies	50 open and 50 laparoscopic nephrectomy. Half of the subjects in eachgroup received ginger essence	Half of the subjects in each group received placebo	Consumption: Before surgery evaluation : every 15 min for the first 2 postoperative h and the 6th hour	Using ginger essence lowered nausea and vomiting In the first 2 postoperative hours, vs. patients with the same surgery but receiving placebo
Zeraati, Shahinfar, Hesari, Masrorniya, & Nasimi (2016)	RCT	Cesarean section spinal anesthesia	25 drops of ginger extract in 30 cc of water	30 cc of water	Consumption: 1 h before surgery evaluation: 2, 4 hr after surgery	Ginger decreased the incidence and severity of nausea and vomiting during the cesarean section no statistically significant relationship at 2, 4 hr after surgery
Seidi, Ebnerasooli, Shahsawari, & Nzarian (2017)	RCT	After cataract surgery under general anesthesia	1‐a ginger capsule in a single 1 g dose 2‐ 2 separate doses of ginger capsule each containing 500 mg	Placebo	Consumption: before operation: 6 AM day of surgery (single dose) or 10 PM (day before and 6 AM day of surgery for 2 dose Evaluation: 6 h after operation	The frequency and intensity of nausea and the frequency of vomiting among those ginger consumers in 2 separate 500 mg doses were less than the one dose and they both were less than placebo
Dabaghzadeh, Khalili, Dashti‐Khavidaki, Abbasian, & Moeinifard (2014)	RCT	Antiretroviral therapy. (HIV + patients)	500 mg ginger (×2)/day	Placebo	Consumption: 30 min before each dose of antiretroviral Regimen for 14 days	Frequency of mild, moderate, and severe nausea and reported at least one episode of vomiting were significantly lower in the ginger than placebo
Emrani, Shojaei, & Khalili (2016)	Pilot RCT	Antituberculosis drug	500 mg ginger/day (2 Zintoma)	Placebo	Consumption one‐half hour before each daily dose of antituberculosis drugs (fasting) for 4 weeks	Nausea was more common in the placebo than the ginger group
Palatty et al. (2013)	Review (3 studies about this topic (2003–2006) (Apariman, Ratchanon, & Wiriyasirivej, 2006; Pongrojpaw & Chiamchanya, 2003; Tavlan et al., 2006)	Outpatient gynecological laparoscopy	0.5 g ginger powder (×2)	Placebo	Consumption: 1 hr before the procedure Evaluation: at 2, 4 and 24 hr after operation	The incidence of the nausea and visual analogue nausea scores was lower in ginger group than placebo at 2 and 4 hr. No difference at 24 hr was found in both groups. Incidence and frequency of vomiting between 2 groups were not statistically different
		Gynecological laparoscopy	0.5 g ginger powder (×3)	Placebo	Consumption: 1 h before the procedure Evaluation: at 2 and 6 h after operation.	At 6 h postoperation, ginger lowered nausea and borderline vomiting but at 2 hr, differences between the two groups for nausea and vomiting were not significant
		General anesthesia for thyroidectomy	0.5 g of ginger+ dexamethasone (IV, immediately before the induction of anesthesia)	Placebo + dexamethasone (IV, immediately before the induction of anesthesia)	Consumption: 1 prior to surgery Evaluation: over 24‐hr after surgery	Dexamethasone plus ginger did not significantly reduce nausea and vomiting compared with dexamethasone during the observation period

Generally, the most common and well‐established use of ginger is its utilization in alleviating symptoms of nausea and vomiting (Lete & Allué, 2016).

Although the included studies generally reported statistically significant reductions in nausea and vomiting measures, the clinical significance of these results was controversial, which could perhaps be explained by the nonstandardized preparations of ginger used and inconsistencies in study methods and outcomes. Multiple potential mechanisms of action have been identified including 5‐HT3 receptor antagonism, anti‐inflammatory properties, and the modulation of gastrointestinal motility (Marx, Kiss, & Isenring, 2015; Marx et al., 2013) (Table 4).

**Table 4:** Motion sickness or sport‐induced nausea and vomiting

Study ID	Type of article	Cause of nausea and vomiting	Intervention (ginger dose per day)	Comparator	Duration	Main results
Palatty et al. (2013)	Review 4 studies about this topic 1982–2003 (Grøntved, Brask, Kambskard, & Hentzer, 1988; Lien et al., 2003; Mowrey & Clayson, 1982; Stewart, Wood, Wood, & Mims, 1991)	Motion sickness by tilted rotating chair	(940 mg Gelatincapsules of ginger powder (×2) +dimenhydrinate	Placebo+ dimenhydrinate	Consumption: 20–25 min before rotating Evaluation: Every 15 s for up to 6 min	Ginger was more effective than dimenhydrinate in reducing experimentally induced motion sickness
		Motion sickness by circular vection	1,000‐ or 2,000‐mg ginger capsules	Placebo	Consumption: 1 hr before circular vection evaluation: at 0, 15, 30, 45, 60, 120, 180, and 240 min after vection cessation	Ginger at a dose of 1,000 mg effectively reduced the severity of nausea, prolonged latency before the onset of nausea and shortening the recovery time from nausea after the cessation of vection. Ginger at a dose of 2,000 mg did not provide further therapeutic effects
		Motion sickness by Head movements in a rotating chair	Ginger 500 mg Ginger 1000 mg 1000 mg fresh ginger	Placebo scopolamine	Subjects made timed head movements in a rotating chair until they reached an end point of motion sickness short vomiting	Neither ginger powder (whole root, 500 or 1000 mg) nor fresh ginger (1000 mg) was effective in altering the gastric function and was also devoid of antimotion sickness but subjects had more head movements with scopolamine than placebo
		Sea sickness in naval cadets unaccustomed to sailing in heavy seas	1 g of powdered ginger	Placebo	Evaluation every hour for 4 consecutive hours after ingestion drug or placebo.	Ginger was observed to be effective in reducing the tendency to vomiting, nausea, vertigo cold, and sweating
Ball et al. (2015)	RCT	Exercise in. recreational athletes	450 ml of beverage A (contained 7.5% glucose, 10 mMNaCl, citric acid, K sorbate and 62.5 ml of ginger root extract per 1 L)	1‐beverage B (beverage A but the ginger was replaced with 62.5 ml of carrot extract) 2‐water	225 ml immediately prior to and 225 ml following exercise	Consuming the beverages did not exacerbate the GI symptoms during exercise. After exercise, the prevalence of stomach problems and nausea decreased with beverage A but neither beverage B nor water

## 4 FATTY LIVER

Nonalcoholic fatty liver disease (NAFLD) is one of chronic liver diseases throughout the world that mostly is seen in obese, low active people, and patients with type II diabetes (Ong & Younossi, 2007). There is no proven medical treatment for this disorder; however, following a healthy life style, modified diet and exercise are suggested by researchers (Thoma, Day, & Trenell, 2012). Previous studies have shown that a diet rich in antioxidants and anti‐inflammatory agents can be effective in the treatment of NAFLD (Eslamparast, Eghtesad, Poustchi, & Hekmatdoost, 2015). Rahimlou, Yari, Hekmatdoost, Alavian, & Keshavarz (2016) conducted the first randomized, double‐blind clinical trial study that examined the effects of ginger supplementation with lifestyle intervention on liver enzymes, inflammatory markers, steatosis, and hepatic fibrosis scores in patients with nonalcoholic fatty liver disease (NAFLD). In this study, 44 patients with NAFLD consumed 2 g of ginger supplement or placebo per day for 12 weeks. In both groups, patients were advised to follow a balanced diet and physical activity program and resulted in a significant decrease in inflammatory marker levels, liver enzymes, hepatic steatosis, and insulin resistance (that is one of the major risk factors in the pathogenesis of NAFLD) in ginger group. Inflammatory cytokines such as TNF‐a play critical role in development of insulin resistance and liver fibrosis and increase fatty acids oxidation. Active compounds in ginger can enhance the antioxidant defense systems such as glutathione peroxidase and glutathione S‐transferase, and reduce levels of malondialdehyde (MDA) and hepatic steatosis. Thus, reduction of TNF‐α and other inflammatory factors resulted in improvement of the NAFLD characteristics. In this study, no significant decrease was observed in liver fibrosis score in the ginger group compared to the placebo group. This can be due to the short term of the intervention, because it takes a long time for regeneration of hepatic tissue and reduction of fibrotic tissue. Further RCTs are needed to find the mechanism of action and ideal dose of ginger supplementation for NAFLD management.

## 5 IRRITABLE BOWEL SYNDROME (IBS)

Irritable bowel syndrome (IBS) is a common functional chronic gastrointestinal disorder, consisting of abdominal discomfort with changes in bowel habits. The effectiveness of current therapeutic strategies for IBS is limited and about 40% of patients use alternative medicine to treat their symptoms (Van Tilburg et al., 2008). Due to the known gastrointestinal effects of ginger, it was reported as the most popular remedy consumed, in a large study of 600 IBS patients (Van Tilburg, Palsson, Ringel, & Whitehead, 2014; Van Tilburg et al., 2008). Van Tilburg et al. (2014) evaluated ginger for IBS treatment in a pilot study of 45 IBS patients, who were randomly consumed placebo, or 1 g of ginger, or 2 g of ginger daily for 28 days. Ginger was well tolerated but it did not show better effects in comparison with placebo. Since this is the first clinical trial on this topic and is a pilot study, it may not be powered enough to find significant results. The investigators also examined trends of these effects, and they found a trend of more improvement of IBS symptoms with placebo (brown sugar) than ginger. The placebo response in this study was 57% with twice as many side effects as the ginger group. In this study, the efficacy of ginger decreased with increase in dosage (26% decrease in symptoms with 1 g versus 12% decrease in symptoms with 2 g of ginger). Optimal ginger dosing should be examined in future trials.

## 6 GASTROENTERITIS

Nonsteroid anti‐inflammatory drugs (NSAIDs) represent as an important and prevalent medicine with common side effects of gastrointestinal (GI) tract (Solomon & Goodson, 2007). (Drozdov, Kim, Tkachenko, & Varvanina (2012) investigated the GI health in 43 patients with confirmed osteoarthritis receiving 1000 mg glucosamine with either ginger (340 mg EV.EXT 35 Zingiber officinalis extract) or Diclofenac (a member of NSAIDs) per day for 4 weeks. In this study, ginger group showed significantly lowered gastrointestinal pain and no change in dyspepsia but esophagogastroduodenoscopy showed significantly increased levels of prostaglandins (PGs) PGE1, PGE2, and PGF2a in the stomach mucosa. This rise in gastric mucosa PG levels correlated with an increase in serum gastrin‐17, whereas Diclofenac group showed increased gastrointestinal pain and dyspepsia with a corresponding significant decrease in stomach mucosa prostaglandins and general negative stomach mucosa degeneration. Both groups showed a relevant and significantly lowered arthritic pain, both on standing and moving, so that ginger was as effective as Diclofenac in reducing pain, while it was safer.

## 7 GASTROINTESTINAL MALIGNANCIES

In a randomized clinical trial, 20 subjects at increased risk for colorectal cancer were assigned to receive either 2 g/day ginger or placebo for 28 days. They showed that ginger was tolerable and safe; however, it did not decrease eicosanoid levels in people at increased risk for colorectal cancer (Zick et al., 2015). Moreover, Zick et al. reported no significant difference in eicosanoids level in 30 subjects at normal risk for colorectal cancer. However, they found a significant decrease in PGE2 and 5‐hydroxyeicosatetraenoicacid (HETE) and a trend toward significant decrease in 12‐HETE and 15‐HETE normalized to free arachidonic acid (Zick et al., 2011). In another trial, the effects of ginger (2 g for 28 days) on apoptosis, proliferation, and differentiation in the normal‐appearing colonic mucosa from 20 patients at increased risk for colorectal cancer showed that ginger supplementation may reduce proliferation in the crypts of normal‐appearing colorectal epithelium, increase apoptosis and differentiation relative to proliferation, especially in the differentiation zone of crypts. This beneficial effect of ginger was found to be associated with downregulation of Bax, human telomerase reverse transcriptase (hTERT), and MIB‐1, while p21 and Bcl‐2 expression remained relatively unchanged. The estimated treatment effect on MIB‐1 was not as strong, the estimated effect was more pronounced in the upper sections of the colorectal crypts, suggesting that ginger may decrease proliferation in the parts of the colorectal crypts most exposed to bowel lumen carcinogens (Citronberg et al., 2013). These findings are consistent with previous studies that suggested that the chemopreventive properties of ginger may be due to regulate cell function and viability (Keum et al., 2002; Lee & Surh, 1998; Miyoshi et al., 2003; Pan et al., 2008). In vitro and animal studies also suggest that ginger and its constituents may act as chemopreventive agents by reducing COX‐2 expression immune function, lowering the activity of microbial enzymes (β‐glucuronidase and mucinase), and blocking angiogenic signals that supply blood to tumor cells (Brown et al., 2009; Khater, 2010; Lu et al., 2011; Seo, Lee, & Kim, 2005; Yagihashi, Miura, & Yagasaki, 2008). Ginger has anti‐inflammatory activities that play roles on its chemopreventive potential against colorectal cancer. In a study on 30 normal participants and 20 participants at increased risk for colorectal cancer, ginger significantly lowered COX‐1 protein expression in participants at increased risk for colorectal cancer but not in the participants at normal risk. However, ginger did not alter 15‐hydroxyprostaglandin dehydrogenase (PGDH) protein expression in either increased or normal risk participants (Jiang et al., 2013). Some findings of other studies about beneficial effects of ginger and its constituents in GI cancer patients included: inhibition of COX and decrease in PGE2 concentrations in colorectal cancer (Levine et al., 2008), reduction in the incidence and multiplicity of adenomas (Zick et al., 2015), decrease in proliferation (hTERT and MIB‐1) and differentiation (p21waf1/cip1) in colonocytes (Stoner, 2013), inhibition of CYP450, 1‐aminobenzotriazole, and aldo‐keto reductase in human liver microsomes, prevention of the formation of M14 and M15 and 18β‐glycyrrhetinic acid in human liver microsomes (Chen, Soroka, Zhu, & Sang, 2013).

## 8 DYSPHAGIA

Dysphagia is a major alarm sign in gastroenterology; however, impaired swallowing function from a combination of underlying disease and reduced physical and cognitive abilities could be a result of elderly. This phenomenon and, in particular, dysphagia are associated with reduced cough reflex leading to increased risk of aspiration pneumonia (Hirata et al., 2016). Furthermore, it may result in malnutrition, dehydration, and decreasing the patient's quality of life. The swallowing reflex is controlled by substance P (SP) that released by nerve endings in the bronchial mucosa and oral cavity (Jin et al., 1994; Ujiie, Sekizawa, Aikawa, & Sasaki, 1993). Reduced salivary SP levels in older people are reported to be lower than in healthy younger individuals (Sekizawa, Ujiie, Itabashi, Sasaki, & Takishima, 1990). Hirata et al. evaluated the application of ginger orally disintegrating (OD) tablets, which prepared by mixing the excipients with the same amount of mannitol and sucrose to a concentration of 1% ginger, on improvement of swallowing function in eighteen healthy older adult aged 63–90. Saliva was collected and endoscopy was performed before and 15 min after ingestion of the placebo and ginger OD tablets. The results showed that 15 min after taking the ginger OD tablets, salivary SP amount was significantly higher than prior to ingestion or after taking the placebo so that it increased near to what observed in healthy young adults. Moreover, no aspiration occurred and a significant improvement in the swallowing function score was observed. Prior to this, they had examined OD tablets on 12 healthy adult male and found SP level of saliva increased immediately after oral ingestion and showed a significantly higher concentration of SP in saliva between 15 and 120 min after oral ingestion as compared to placebo. The mechanism responsible for improving swallowing function is believed to be a result of the gingerol and shogaol components of ginger that act as a TRPV1 agonist (Abe et al., 2015; Hirata et al., 2016). Krival & Bates (2012) measured differences in peak lingua‐palatal swallowing pressures, pressure durations, and pressure adjustments in response to two volumes of water and carbonation (in Schweppes_ Club Soda) and carbonation+gingerol (in Reed's Extra Ginger Brew) in 20 young adult women. The study showed that stimulus on lingua‐palatal swallowing pressure and rising and releasing lingua‐palatal pressure duration were greater for carbonation + gingerol and carbonation than for water.

## 9 ADVERSE EFFECTS AFTER INGESTION OF GINGER

Ginger consumption rarely induces side effects such as mild gastrointestinal complications such as heartburn, belching, bruising or flushing, rash, and gastrointestinal discomfort. Adverse events were generally not significantly higher in the ginger group compared to the control group (Lete & Allué, 2016; Marx et al., 2013). In a study of 27 healthy volunteers who consumed a single oral dose of 100 mg to 2 g ginger, minor gastrointestinal upset was the major adverse effect (Zick et al., 2009). Despite previous studies indicating that ginger could interfere with platelet aggregation and cause excessive bleeding (Srivastava, 1986), a recent crossover study of 12 healthy volunteers who consumed 1.2 g of dried ginger rhizome three times per day for 2 weeks, ginger did not affect platelet aggregation and had no effect on the pharmacokinetics or pharmacodynamics of a single 25 mg dose of warfarin taken on day 7 (Jiang et al., 2005). Viljoen et al. (2014) also reviewed the safety of ginger as a secondary objective in their meta‐analysis of nausea and vomiting of pregnancy and found that ginger did not have a risk for side effects or adverse events during pregnancy. In a prospective study, the pregnancy outcome of 187 women who were exposed to ginger during the first trimester of pregnancy was compared with women who had been exposed to non‐teratogenic drugs that were not antiemetic. There were no statistically significant differences between the two groups in terms of live births spontaneous abortions, therapeutic abortions, birth weight, or gestational age (Portnoi et al., 2003). A larger population‐based cohort study in Norway (68,522 women) showed that the use of ginger during pregnancy (1020 women, 1.5%) was not associated with an increased risk of congenital malformations, still birth/perinatal birth, low birth weight, or preterm birth (Heitmann, Nordeng, & Holst, 2013).

## 10 CONCLUSION

Based on evidence from this systematic review, ginger could be considered a harmless and possibly effective alternative option for women suffering from the symptoms of nausea and vomiting in pregnancy. It seems that divided lower daily dosage of 1500 mg ginger is beneficial for nausea relief. Ginger did not pose a risk for side effects or adverse events during pregnancy. Ginger and its polyphenols have been shown to target multiple signaling molecules that provide a basis for its use against multifactorial human diseases such as cancer. Moreover, most of the known activities of ginger components are based only on in vitro and in vivo studies, except for a few clinical studies in some gastrointestinal disorders especially nausea and vomiting in human subjects and limited number in some other complications that may not be as much powered as to find significant results. Therefore, more extensive and well‐controlled human studies are required to demonstrate its efficacy as gastroprotective agent, as it is a safe and cost‐effective alternative. Dose‐finding studies should be undertaken to accurately determine the effective dose and preparation of ginger.

## FUNDING

There is no funding associated with this study.

The work has done at Department of Clinical Nutrition and Dietetics, Faculty of Nutrition Sciences and Food Technology, National Nutrition and Food Technology Research Institute, Shahid Beheshti University of Medical Sciences, Tehran, Iran.

## ETHICAL STATEMENT

This study does not involve any human or animal testing.

[Correction added on November 23, 2018 after online publication: the Ethical Statement was changed from “Since it.” to “This study does not involve any human or animal testing.”.]

## CONFLICT OF INTEREST

The authors declare there is no conflict of interest.
